# 

**Published:** 1868-05-01

**Authors:** 


					﻿CONTENTS :
Progressive Paralysis of the Insane. By A. W. Bosworth, M.A., Paris,
France....................................................... 283
Foreign Correspondence :
Lettej^rom Samuel Cole, M.D., Heidelberg....................  300
Editoria'tT:	X,
Items of News and Gossip...................................... 308
Richmond (Va.) Medical Journal............................... 3I2
Illinois StateMfedical Society............................... 3I2
Duties of thc-Wedical Profession.............................
Errata....................................................... ^13
Society Reports:
Chicago Medical Society.. . .	  314
Champaign County Medical Society............................. 314
The Brainard Medical Society................................. 314
				

